# ICU admissions and in-hospital deaths linked to COVID-19 in the Paris region are correlated with previously observed ambient temperature

**DOI:** 10.1371/journal.pone.0242268

**Published:** 2020-11-20

**Authors:** Mehdi Mejdoubi, Xavier Kyndt, Mehdi Djennaoui

**Affiliations:** 1 Department of Radiology, Hospital of Valenciennes, Valenciennes, France; 2 Department of Public Health, Hospital of Valenciennes, Valenciennes, France; Erasmus Medical Center, NETHERLANDS

## Abstract

The purpose of this ecological study was to explore the association of weather with severity indicators of coronavirus disease 2019 (COVID-19). Daily COVID-19-related intensive care unit (ICU) admissions and in-hospital deaths in the Paris region and the daily weather characteristics of Paris midtown were correlated with a time lag. We assessed different study periods (41, 45, 50, 55, and 62 days) beginning from 31 March 2020. Daily ICU admissions and in-hospital deaths were strongly and negatively correlated to ambient temperatures (minimal, average, and maximal). The highest Pearson correlation coefficients and statistically significant p values were found 8 days before the occurrence of ICU admissions and 15 days before deaths. Partial correlations with adjustment on days since lockdown showed similar significant results. The study findings show a negative correlation of previously observed ambient temperature with severity indicators of COVID-19 that could partly explain the death toll discrepancies between and within countries.

## Introduction

The ongoing major epidemic of coronavirus disease 2019 (COVID-19) infection began in cold areas of temperate countries of the northern hemisphere [1], which raises the question of weather influence.

Continental France has been severely hit by this outbreak of COVID-19, with around 30 000 deaths, including more than 19 000 in-hospital deaths (as of 15 June 2020). The main affected regions were its eastern region (mainly Alsace) and the Paris region (Île-de-France), with more than 3500 and 7200 in-hospital deaths, respectively.

Monitoring the outbreak of COVID-19 with biological testing is imprecise as the infection can be asymptomatic [2]. Data such as deaths or intensive care unit (ICU) admissions (reflecting critically ill patients) are more reliable, especially in highly developed countries. In the natural course of infection with COVID-19, a pulmonary worsening may appear around 5 to 9 days after infection onset [2, 3], with an immunological mechanism known as “cytokine storm” [1] that may lead to hospitalization. Subsequently, death may occur at different moments (depending on hospitalization, ICU admission, and ventilation). The median duration of hospitalization before death is around 9 days [3] in COVID-19 critically ill patients in a highly developed country, therefore death should occur around 14 to 18 days after the onset of infection, although it can of course happen much later. A weather effect, if one exists, could influence a patient’s ICU admission or death on the day of occurrence or during the days beforehand. Once a patient has been hospitalized, no weather effect should be seen in highly developed countries if hospital ambient air is regulated (which is the case in France).

To study the hypothesis of a weather effect on severity indicators of COVID-19, we tested the correlation between these indicators and weather characteristics in a single area with a high-density population and a heavy COVID-19 burden. We hypothesized that colder temperatures are responsible for a higher hospitalization rate and a higher mortality rate, with a delay due to the course of evolution of this pathology.

## Methods

### Setting

The Paris region (Île-de-France) has a population of 12 million inhabitants, with the highest population density in France (mainly located in Paris and its suburbs within a 30-km range). The Paris region was chosen as it has the highest death toll in France and is the smallest region (12,012 km^2^). The weather is therefore rather homogeneous throughout the region and can be approximated by the weather in midtown Paris. The healthcare system is characterized by a high density of public and private hospitals with no restriction on access to healthcare. The capacity of ICU beds in France was doubled by the end of March 2020, and there was no significant shortage of this kind of bed during the outbreak. Prospectively monitored data (ICU admissions, in-hospital deaths) for COVID-19 infection in the Paris region, as everywhere in France, are electronically centralized. A lockdown in France was started on 17 March 2020, and the peak number of deaths was reached on 6 April 2020. A progressive but significant reduction in cases and deaths began after the first week of May. Lockdown was eased on 11 May, with mandatory rules on social distancing.

### Data sources

Daily hospital data (COVID-19-related in-hospital deaths, ICU admissions) in the Paris region were retrieved from the national governmental database “Geodes” [4]. Meteorological factors (hours of sunshine, rainfall, minimal temperature, maximal temperature) were retrieved from the French national meteorology agency [5]. Mean temperature was estimated using the formula:

$$\text{Mean}\;\text{temperature}=\frac{\left(\text{minimal}\;\text{temperature}+\text{maximal}\;\text{temperature}\right)}{2}$$

All of these data are public and freely accessible.

### Statistical analysis

**Fig 1:**
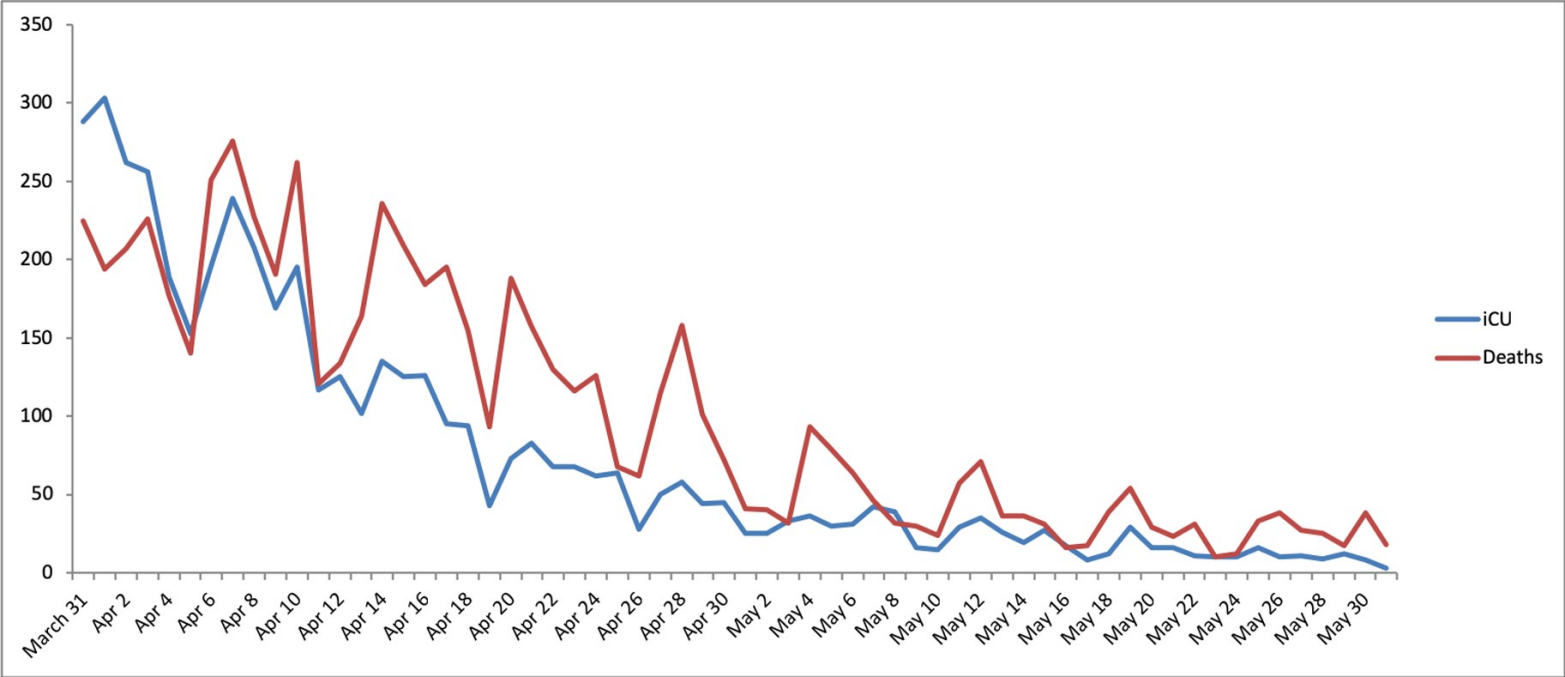
Paris region daily COVID-19 ICU admissions and in-hospital deaths curves (period: 31 March to 30 May 2020).

Meteorological factors of Paris midtown were correlated against hospital data on the same day, as death or ICU admission can happen on arrival at the hospital. As weather may have influenced the prognosis of patients before hospitalization, we sequentially tested daily hospital data with the weather observed from 0 to 19 day(s) before ICU admission or death. The period from 31 March to 10 May 2020 was chosen because it corresponds to stabilization of the outbreak following the national lockdown. During this 41-day period, there were 4,355 ICU admissions and 5,641 deaths in the Paris region (Fig 1). Weather characteristics were therefore observed from 12 March until 10 May. We also assessed longer periods (45, 50, 55, and 62 days), with hospital data beginning from 31 March and weather data from 12 March to 31 May (there were totals of 4,689 ICU admissions and 6,299 deaths for the longest period).

A bivariate analysis was performed and Pearson’s correlation coefficients calculated. In order to consider the influence of lockdown on ICU admissions and deaths, we completed the analysis by partial correlation, which allowed adjusting Pearson’s correlation on days since lockdown. Considering the number of statistical tests performed (20 tests, from 0 to 19 days), we applied Bonferroni’s correction on significance level, which was set at 0.25%. A correlation coefficient >0.7 was considered as very strong, 0.7 to 0.5 as strong, 0.5 to 0.3 as mild, and <0.30 as weak.

The statistical analyses were performed with R software version V4.0.0. For partial correlation, we used R package ppcor [6].

### Patient and public involvement

No patients were directly involved in this study.

## Results

**Fig 2:**
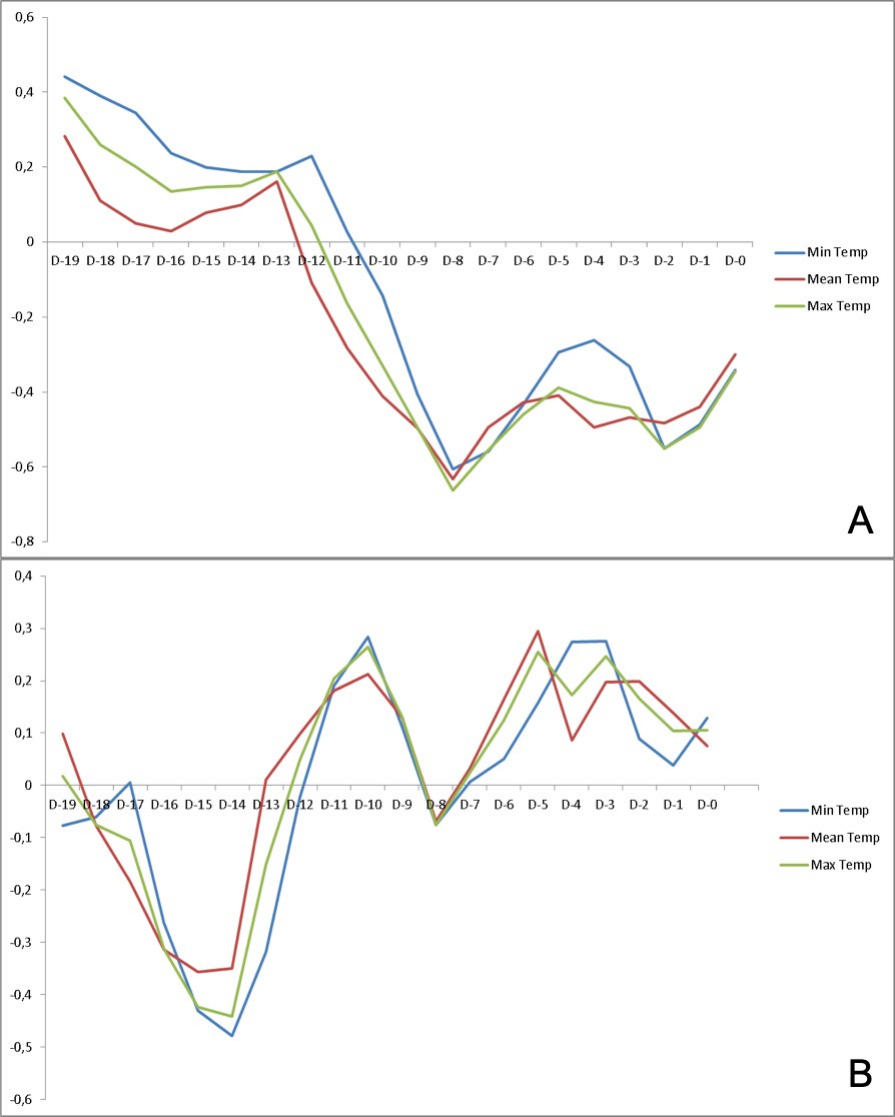
Partial correlation coefficients (adjusted on days since lockdown) of midtown Paris temperatures and (A) daily ICU admissions and (B) in-hospital deaths in the Paris region. In ordinate is partial correlation coefficient (0 to –1), in abscissa (days [D] –19 to 0).

No statistical correlations were found between sunshine or rain and ICU admissions or in-hospital deaths. There was a negative correlation with minimal, mean, and maximal temperatures with both outcomes. This correlation was considered strong/very strong (with all three temperatures) for ICU admissions from day –12 to day –6, with the highest correlation at day –8. This correlation was considered strong/very strong for in-hospital deaths from day –16 to day –11 (with all three temperatures) with the highest correlation at day –15. Correlations with temperature were statistically significant for most of the considered period for ICU admissions (Table 1) and in-hospital deaths (Table 2). Partial correlation (Fig 2) showed a strong negative, significant correlation at day –8 for ICU admissions (with all three temperatures) and a second, smaller peak at day –2 (Table 3). There was a mild negative, significant correlation at day –14 (only for minimal temperature) for in-hospital deaths (Table 4).

**Table 1:** Correlations between COVID-19-related ICU admissions in the Paris region and temperatures measured from 19 days before ICU admission to the date of admission (study period: 31 March to 10 May 2020).

Day	Minimal observed temperature		Mean calculated temperature		Maximal observed temperature	
	Correlation coefficient	p value	Correlation coefficient	p value	Correlation coefficient	p value
−19	−0.246	0.121	−0.430	0.005	−0.523	<0.0025
−18	−0.311	0.048	−0.493	<0.0025	−0.582	<0.0025
−17	−0.333	0.034	−0.520	<0.0025	−0.609	<0.0025
−16	−0.409	0.008	−0.554	<0.0025	−0.615	<0.0025
−15	−0.451	0.003	−0.570	<0.0025	−0.610	<0.0025
−14	−0.449	0.003	−0.552	<0.0025	−0.586	<0.0025
−13	−0.471	<0.0025	−0.561	<0.0025	−0.583	<0.0025
−12	−0.528	<0.0025	−0.631	<0.0025	−0.656	<0.0025
−11	−0.609	<0.0025	−0.699	<0.0025	−0.705	<0.0025
−10	−0.685	<0.0025	−0.733	<0.0025	−0.706	<0.0025
−9	−0.752	<0.0025	−0.749	<0.0025	−0.685	<0.0025
−8	−0.789	<0.0025	−0.767	<0.0025	−0.687	<0.0025
−7	−0.755	<0.0025	−0.709	<0.0025	−0.611	<0.0025
−6	−0.703	<0.0025	−0.657	<0.0025	−0.561	<0.0025
−5	−0.632	<0.0025	−0.581	<0.0025	−0.482	<0.0025
−4	−0.604	<0.0025	−0.568	<0.0025	−0.478	<0.0025
−3	−0.590	<0.0025	−0.546	<0.0025	−0.449	0.003
−2	−0.655	<0.0025	−0.589	<0.0025	−0.472	<0.0025
−1	−0.641	<0.0025	−0.557	<0.0025	−0.420	0.006
0	−0.569	<0.0025	−0.465	<0.0025	−0.313	0.046

ICU, intensive care unit.

**Table 2:** Correlations between in-hospital COVID-19-related deaths in the Paris region and temperatures measured from 19 days before death to the date of death (study period: 31 March to 10 May 2020).

Day	Minimal observed temperature		Mean calculated temperature		Maximal observed temperature	
	Correlation coefficient	p value	Correlation coefficient	p value	Correlation coefficient	p value
−19	−0.414	0.007	−0.508	<0.0025	−0.527	<0.0025
−18	−0.445	0.004	−0.566	<0.0025	−0.603	<0.0025
−17	−0.420	0.006	−0.585	<0.0025	−0.652	<0.0025
−16	−0.572	<0.0025	−0.679	<0.0025	−0.701	<0.0025
−15	−0.668	<0.0025	−0.736	<0.0025	−0.724	<0.0025
−14	−0.684	<0.0025	−0.731	<0.0025	−0.708	<0.0025
−13	−0.631	<0.0025	−0.633	<0.0025	−0.583	<0.0025
−12	−0.566	<0.0025	−0.581	<0.0025	−0.544	<0.0025
−11	−0.502	<0.0025	−0.533	<0.0025	−0.510	<0.0025
−10	−0.496	<0.0025	−0.495	<0.0025	−0.452	0.003
−9	−0.549	<0.0025	−0.505	<0.0025	−0.429	0.005
−8	−0.594	<0.0025	−0.548	<0.0025	−0.467	<0.0025
−7	−0.539	<0.0025	−0.480	<0.0025	−0.391	0.011
−6	−0.507	<0.0025	−0.413	0.007	−0.301	0.056
−5	−0.432	0.005	−0.297	0.059	−0.163	0.309
−4	−0.364	0.019	−0.306	0.052	−0.227	0.154
−3	−0.321	0.041	−0.242	0.128	−0.153	0.34
−2	−0.392	0.011	−0.283	0.073	−0.169	0.292
−1	−0.424	0.006	−0.300	0.056	−0.163	0.308
0	−0.360	0.021	−0.261	0.099	−0.143	0.372

**Table 3:** Correlations between COVID-19-related ICU admissions in the Paris region and temperatures measured from 19 days before ICU admission to the date of admission (study period: 31 March to 10 May 2020)–partial correlations adjusted on days since lockdown.

Day	Minimal observed temperature		Mean calculated temperature		Maximal observed temperature	
	Correlation coefficient	p value	Correlation coefficient	p value	Correlation coefficient	p value
−19	0.442	0.004	0.283	0.077	0.384	0.014
−18	0.391	0.013	0.111	0.494	0.259	0.107
−17	0.345	0.029	0.05	0.758	0.201	0.214
−16	0.238	0.139	0.03	0.852	0.136	0.403
−15	0.200	0.215	0.079	0.627	0.146	0.367
−14	0.189	0.243	0.099	0.545	0.151	0.352
−13	0.188	0.246	0.162	0.318	0.188	0.245
−12	0.229	0.155	−0.108	0.508	0.045	0.785
−11	0.027	0.867	−0.282	0.078	−0.163	0.314
−10	−0.143	0.379	−0.411	0.008	−0.330	0.037
−9	−0.405	0.01	−0.497	<0.0025	−0.494	<0.0025
−8	−0.606	<0.0025	−0.633	<0.0025	−0.663	<0.0025
−7	−0.559	<0.0025	−0.494	<0.0025	−0.555	<0.0025
−6	−0.434	0.005	−0.429	0.006	−0.460	0.003
−5	−0.295	0.065	−0.410	0.009	−0.389	0.013
−4	−0.261	0.104	−0.494	<0.0025	−0.427	0.006
−3	−0.331	0.037	−0.468	<0.0025	−0.443	0.004
−2	−0.552	<0.0025	−0.484	<0.0025	−0.551	<0.0025
−1	−0.487	<0.0025	−0.439	0.005	−0.495	<0.0025
0	−0.341	0.031	−0.300	0.06	−0.345	0.029

ICU, intensive care unit.

**Table 4:** Correlations between in-hospital COVID-19-related deaths in the Paris region and temperatures measured from 19 days before death to the date of death (study period: 31 March to 10 May 2020)–partial correlations adjusted on days since lockdown.

Day	Minimal observed temperature		Mean calculated temperature		Maximal observed temperature	
	Correlation coefficient	p value	Correlation coefficient	p value	Correlation coefficient	p value
−19	−0.077	0.635	0.099	0.544	0.018	0.91
−18	−0.06	0.713	−0.077	0.637	−0.075	0.648
−17	0.005	0.977	−0.184	0.255	−0.105	0.52
−16	−0.263	0.102	−0.314	0.049	−0.313	0.049
−15	−0.431	0.006	−0.356	0.024	−0.423	0.006
−14	−0.479	<0.0025	−0.349	0.027	−0.441	0.004
−13	−0.318	0.046	0.011	0.945	−0.151	0.354
−12	−0.022	0.891	0.098	0.548	0.049	0.763
−11	0.191	0.239	0.181	0.265	0.204	0.206
−10	0.283	0.077	0.213	0.188	0.265	0.099
−9	0.113	0.488	0.130	0.425	0.132	0.416
−8	−0.075	0.647	−0.069	0.67	−0.076	0.64
−7	0.007	0.965	0.033	0.838	0.024	0.883
−6	0.050	0.761	0.164	0.313	0.125	0.443
−5	0.157	0.333	0.294	0.066	0.255	0.112
−4	0.274	0.087	0.086	0.598	0.173	0.286
−3	0.276	0.085	0.197	0.224	0.246	0.126
−2	0.089	0.586	0.198	0.22	0.166	0.307
−1	0.038	0.815	0.139	0.392	0.104	0.522
0	0.129	0.427	0.075	0.646	0.106	0.515

**Fig 3:**
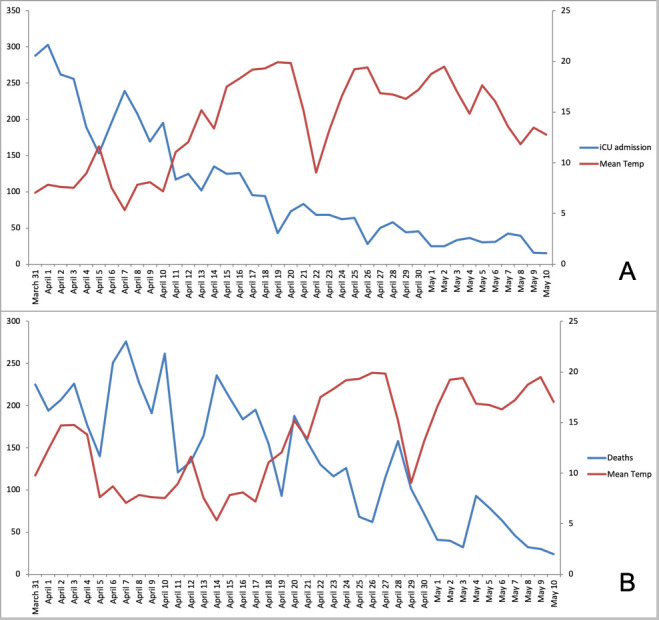
Evolution of (A) ICU admissions and (B) daily death tolls (study period: 31 March to 10 May 2020) superimposed with mean environmental temperatures observed 8 days before ICU admission and 15 days before death (as these showed the highest correlation coefficients). Left ordinate is daily toll (0–350), right ordinate is temperature (0–25°C).

A temperature drop preceded the peak of ICU admissions and in-hospital deaths (Fig 3), with the highest correlation coefficients corresponding to time lags of 8 and 14–15 days, respectively.

The longer study periods 45, 50, 55 days (data not shown) and 62 days (Tables 5–8) also showed a negative correlation between severity indicators and temperature (maximal correlation coefficients at around –14 to –15 days for deaths and –8 days for ICU admissions) with significant p values and similar or weaker correlation coefficients.

**Table 5:** Correlations between COVID-19-related ICU admissions in the Paris region and temperatures measured from 19 days before ICU admission to the date of admission (study period: 31 March to 31 May 2020).

Day	Minimal observed temperature		Mean calculated temperature		Maximal observed temperature	
	Correlation coefficient	p value	Correlation coefficient	p value	Correlation coefficient	p value
−19	−0.376	0.00259	−0.492	<0.0025	−0.528	<0.0025
−18	−0.399	<0.0025	−0.513	<0.0025	−0.547	<0.0025
−17	−0.383	<0.0025	−0.503	<0.0025	−0.543	<0.0025
−16	−0.406	<0.0025	−0.504	<0.0025	−0.528	<0.0025
−15	−0.417	<0.0025	−0.504	<0.0025	−0.519	<0.0025
−14	−0.421	<0.0025	−0.491	<0.0025	−0.496	<0.0025
−13	−0.443	<0.0025	−0.493	<0.0025	−0.483	<0.0025
−12	−0.481	<0.0025	−0.547	<0.0025	−0.546	<0.0025
−11	−0.543	<0.0025	−0.609	<0.0025	−0.605	<0.0025
−10	−0.607	<0.0025	−0.651	<0.0025	−0.630	<0.0025
−9	−0.658	<0.0025	−0.663	<0.0025	−0.615	<0.0025
−8	−0.695	<0.0025	−0.683	<0.0025	−0.617	<0.0025
−7	−0.657	<0.0025	−0.629	<0.0025	−0.553	<0.0025
−6	−0.603	<0.0025	−0.581	<0.0025	−0.513	<0.0025
−5	−0.549	<0.0025	−0.536	<0.0025	−0.479	<0.0025
−4	−0.525	<0.0025	−0.522	<0.0025	−0.474	<0.0025
−3	−0.509	<0.0025	−0.501	<0.0025	−0.448	<0.0025
−2	−0.550	<0.0025	−0.527	<0.0025	−0.461	<0.0025
−1	−0.536	<0.0025	−0.496	<0.0025	−0.417	<0.0025
0	−0.467	<0.0025	−0.416	<0.0025	−0.332	0.00841

ICU, intensive care unit.

**Table 6:** Correlations between in-hospital COVID-19-related deaths in the Paris region and temperatures measured from 19 days before death to the date of death (study period: 31 March to 31 May 2020).

Day	Minimal observed temperature		Mean calculated temperature		Maximal observed temperature	
	Correlation coefficient	p value	Correlation coefficient	p value	Correlation coefficient	p value
−19	−0.509	<0.0025	−0.550	<0.0025	−0.525	<0.0025
−18	−0.499	<0.0025	−0.552	<0.0025	−0.538	<0.0025
−17	−0.440	<0.0025	−0.528	<0.0025	−0.540	<0.0025
−16	−0.504	<0.0025	−0.563	<0.0025	−0.550	<0.0025
−15	−0.553	<0.0025	−0.591	<0.0025	−0.558	<0.0025
−14	−0.566	<0.0025	−0.590	<0.0025	−0.548	<0.0025
−13	−0.532	<0.0025	−0.514	<0.0025	−0.450	<0.0025
−12	−0.485	<0.0025	−0.479	<0.0025	−0.428	<0.0025
−11	−0.451	<0.0025	−0.465	<0.0025	−0.433	<0.0025
−10	−0.457	<0.0025	−0.463	<0.0025	−0.429	<0.0025
−9	−0.496	<0.0025	−0.476	<0.0025	−0.423	<0.0025
−8	−0.542	<0.0025	−0.517	<0.0025	−0.455	<0.0025
−7	−0.509	<0.0025	−0.475	<0.0025	−0.407	<0.0025
−6	−0.464	<0.0025	−0.415	<0.0025	−0.339	0.007
−5	−0.407	<0.0025	−0.346	0.00588	−0.267	0.0359
−4	−0.359	0.00421	−0.347	0.00574	−0.307	0.0153
−3	−0.329	0.00907	−0.308	0.015	−0.263	0.0391
−2	−0.372	0.00291	−0.329	0.00895	−0.265	0.037
−1	−0.377	0.00255	−0.326	0.00976	−0.254	0.0467
0	−0.320	0.0112	−0.287	0.0235	−0.231	0.071

**Table 7:** Correlations between COVID-19-related ICU admissions in the Paris region and temperatures measured from 19 days before ICU admission to the date of admission (study period: 31 March to 31 May 2020)–partial correlations adjusted on days since lockdown.

Day	Minimal observed temperature		Mean calculated temperature		Maximal observed temperature	
	Correlation coefficient	p value	Correlation coefficient	p value	Correlation coefficient	p value
−19	0.130	0.3174	−0.169	0.1926	−0.042	0.7452
−18	0.045	0.7308	−0.275	0.0323	−0.147	0.2579
−17	−0.017	0.8965	−0.342	0.0069	−0.216	0.0946
−16	−0.132	0.3089	−0.364	0.0039	−0.282	0.0277
−15	−0.187	0.1480	−0.351	0.0056	−0.299	0.0190
−14	−0.204	0.1150	−0.338	0.0078	−0.298	0.0196
−13	−0.215	0.0964	−0.298	0.0196	−0.280	0.029
−12	−0.234	0.0698	−0.397	<0.0025	−0.348	0.0059
−11	−0.321	0.0115	−0.446	<0.0025	−0.419	<0.0025
−10	−0.379	0.0026	−0.456	<0.0025	−0.451	<0.0025
−9	−0.457	<0.0025	−0.441	<0.0025	−0.475	<0.0025
−8	−0.553	<0.0025	−0.504	<0.0025	−0.557	<0.0025
−7	−0.513	<0.0025	−0.413	<0.0025	−0.485	<0.0025
−6	−0.427	<0.0025	−0.343	0.0069	−0.403	<0.0025
−5	−0.327	0.0100	−0.280	0.0291	−0.319	0.0122
−4	−0.282	0.0275	−0.289	0.0239	−0.304	0.0172
−3	−0.276	0.0316	−0.255	0.0471	−0.281	0.0282
−2	−0.376	0.0029	−0.276	0.0316	−0.34	0.0074
−1	−0.367	0.0036	−0.222	0.0858	−0.305	0.0170
0	−0.256	0.0467	−0.102	0.4335	−0.182	0.1613

ICU, intensive care unit.

**Table 8:** Correlations between in-hospital COVID-19-related deaths in the Paris region and temperatures measured from 19 days before death to the date of death (study period: 31 March to 31 May 2020)–partial correlations adjusted on days since lockdown.

Day	Minimal observed temperature		Mean calculated temperature		Maximal observed temperature	
	Correlation coefficient	p value	Correlation coefficient	p value	Correlation coefficient	p value
−19	−0.166	0.202	−0.148	0.2538	−0.170	0.1914
−18	−0.174	0.1803	−0.247	0.0546	−0.233	0.0705
−17	−0.135	0.299	−0.340	0.0073	−0.270	0.0351
−16	−0.356	0.0048	−0.425	<0.0025	−0.425	<0.0025
−15	−0.504	<0.0025	−0.452	<0.0025	−0.511	<0.0025
−14	−0.542	<0.0025	−0.470	<0.0025	−0.538	<0.0025
−13	−0.422	<0.0025	−0.224	0.0831	−0.334	0.0086
−12	−0.242	0.0602	−0.125	0.3379	−0.189	0.1451
−11	−0.101	0.4369	−0.035	0.7866	−0.069	0.5976
−10	−0.005	0.9673	0.038	0.7711	0.021	0.875
−9	−0.049	0.7061	0.027	0.8353	−0.007	0.958
−8	−0.177	0.1727	−0.127	0.3288	−0.158	0.2237
−7	−0.155	0.2316	−0.078	0.5496	−0.119	0.361
−6	−0.093	0.4778	0.063	0.6269	−0.005	0.9676
−5	0.018	0.8909	0.220	0.0881	0.140	0.2813
−4	0.126	0.334	0.100	0.4423	0.118	0.3632
−3	0.160	0.2194	0.178	0.1702	0.181	0.1633
−2	0.047	0.7205	0.180	0.1648	0.130	0.3196
−1	0.006	0.9607	0.158	0.2226	0.097	0.4565
0	0.091	0.4853	0.137	0.2938	0.125	0.338

For the longest period of 62 days, partial correlation (adjusted on days since lockdown) still showed a strong and significant correlation between temperature and ICU admissions, with the highest correlation coefficient at day –8 (Table 7). For in-hospital deaths, all three temperatures at day –14 were significant, with a strong or mild correlation coefficients (Table 8).

## Discussion

The findings of this study suggest that severity indicators of the COVID-19 outbreak strongly correlated with ambient temperatures in the Paris region. This finding was consistent during different periods of time (41, 45, 50, 55, and 62 days). The first 41-day period corresponded to the stabilization of the outbreak (2 weeks after beginning the national lockdown) and had the highest burden. As time passed, the daily death toll declined sharply.

France has a mainly oceanic climate, which is shared by most of Western Europe (UK, Belgium, Netherlands, and Germany) and the course of this outbreak was shared by the countries of this geographical area (declining during summer and resuming in October). Many early studies [7–10], from different continents and settings, have linked severe acute respiratory syndrome coronavirus 2 (SARS-Cov-2) transmission to meteorological factors. Each 1°C increase in average ambient temperature and in diurnal temperature range reduced the number of daily cases in China [7], whereas a specific absolute humidity of around 6 g/m^3^ favored transmission. Prata et al. [8] described, in a tropical setting, a significant linear negative relationship between confirmed cases of COVID-19 and ambient temperature from 16.8°C to 25.8°C (with a threshold above 25.8°C where the infection curve became flat). Also, the epidemic intensity decreased following days at a higher temperature (with a time lag) in China, and the peak of infections was at approximately 10°C [9]. This finding does not necessarily suggest that lower temperature *per se* affects the virus, as it also modifies human activities. The interactions between ambient temperature, humidity, wind, and hours of sunshine are complex, and are not the purpose of this article. Sajadi et al. [10] found that relative humidity (44% to 84%) and mean temperature (5°C to 11°C) before the first deaths were similar in February in eight different cities (which later became the main hotspots of the outbreak at that time). Ahmadi et al. [11] showed that population density, population movement, and days of infection have a direct relationship with the infection rate in Iran. Wind speed was found to be significantly and inversely correlated to COVID-19 infection rate (i.e. higher wind speed resulted in lower infection rate). Bukhari et al. [12] studied 19 countries and different states within the US, and found that the number of new cases was highest in regions with a mean temperature between 0°C and 17°C and absolute humidity between 1 and 9 g/m^3^. Finally, Pirouz et al. [13] assessed the effect of climatic parameters (average temperature, relative humidity, wind speed) in three regions of Italy (Lombardy, Veneto, Emilia-Romagna) on daily confirmed infections over a period of 1 month. They found a significant negative correlation with average temperature, relative humidity, and wind speed, with a delay of 4–8 days between the impact of weather parameters and the new confirmed cases (which is consistent with the natural course of this pathology). Most of these studies, however, relied on biologically confirmed infections, which are known to depend widely on the biological testing policy and the capability. In France, as elsewhere, biological testing was very limited at the beginning of the pandemic, and only approximately 200,000 patients were confirmed positive, whereas between 2 and 6 million patients were likely to have been infected [14].

Kodera et al. [15] studied infection cases/deaths and weather parameters (averaged over the total duration of the study periods), adjusted to population density and the percentage of elderly people (partial correlation), in 16 Japanese prefectures. They observed lower morbidity/mortality rates for higher temperature and absolute humidity (but no effect of wind velocity and daylight hours). Ma et al. [16], in a study from Wuhan, reported a significant negative association with ambient temperature and absolute humidity on daily deaths from COVID-19. However, although they tested a lagged effect, the maximum lag was only 5 days between the environmental factors tested and the day of death. This does not fit with the course of pathology of this disease, as death often occurs later (and much later in case of hospitalization, which extends patient survival, at least in highly developed countries). They did not find any effect of air pollutants on death, but some authors hypothesized that pollution could favor spread of the virus [17]. Death monitoring may also be imprecise, but is quite reliable in highly developed countries and we sought to work on reliable data from France. With regards to the death toll, we chose to focus on in-hospital deaths, which are more precise for the date of occurrence and the cause. Hospital admission thresholds differ among countries, and depend on hospital beds per capita, ventilator availability, and access to healthcare. Within a country, it depends on patient categorization (mainly age and disease severity). In France, the mean age of hospitalized COVID-19 patients was 68 years and the mean age of the deceased was 79 years [14]. Overall, 18.1% of hospitalized patients died with a fatality ratio depending mainly on patient age (0.001% if <20 years old, 8.3% if ≥80 years), similar to other developed countries. Different subgroups of fatal cases have been identified [14], which is similar to other developed countries like the US [3]. Consistent with this, we found different day peaks of ICU admission and death, consistent with the course of hospitalization and the different types of patients.

Between 27.4% and 44.1% of hospitalized patients with COVID-19 in the US are admitted to the ICU (with or without mechanical ventilation) [18]. Some critically ill patients are not even admitted to the ICU for ventilation therapy, which is often a single “yes or no” decision. We found that ambient temperatures at day –8 correlated most strongly with ICU admissions, whereas temperatures at day –15 correlated most closely with in-hospital deaths. The difference of 7 days corresponds to the median time between ICU admission and in-hospital death in the Paris region and is consistent with the median duration of hospitalization prior to death observed in France [14].

Chin et al. showed that SARS-CoV-2, in in-vitro/lab-based conditions, was highly stable at 4°C, but sensitive to heat [19]. Humidity and temperature influence survival after infection with influenza virus, SARS-CoV, and MERS-CoV, as well as droplet stabilization, propagation in nasal mucosa, human immunity [10, 20], and human way of life (i.e. indoor and outdoor activities). Therefore, a respiratory outbreak has multifactorial drivers and the number and importance of each of these factors is not only unknown, but may vary between countries and climes.

Models show that, while climate may influence this outbreak and/or its severity in a particular location, other factors such as population immunity [20] are crucial for a potential seasonality. Also, public health controls (school closures, restricting mass gatherings, social distancing) play a major role and likely have a greater effect than climate [21].

Although our results clearly indicate a link between temperature and severity indicators of COVID-19, our study does not provide clear answers to the precise effect of temperature. It may be responsible for a greater number of infections that eventually lead to a greater number of deaths, or it may only be responsible for more severe infections, or it may affect both.

### Strengths and limitations

Our study has several strengths. We were able to study reliable data as in-hospital deaths are electronically centralized in France. Lockdown was uniformly applied in France, and was respected by the population, allowing a period of stabilization in April and May. Our study also has some limitations. First, we chose not to study the effect of humidity, which varies more than temperature (between 40% and 90%), depending on the location within the Paris region. Second, the study period is limited to 2 months, but the first wave of this outbreak in France did not allow a longer study period. Finally, our conclusions are valid mainly for temperate countries of the northern hemisphere with a highly developed and universal health system.

## Conclusions

Across Europe and the rest of the world, several factors potentially contribute to the severity of this pandemic. Our findings suggest that weather may contribute to the rise and fall of this outbreak in temperate countries of the northern hemisphere and this could indicate a likely resurgence next winter. Empirically, we observed a lull in COVID mortality during summer in western Europe and a resumption since October. Therefore, social distancing should be maintained on a long-term basis. Temperature is a driver of this outbreak and may influence both viral spread and case fatality.
